# Supplementary material for: “Should I Inhale?”—Perceptions, Barriers, and Drivers for Medicinal Cannabis Use amongst Australian Women with Primary Dysmenorrhoea: A Qualitative Study
Source: Int J Environ Res Public Health. 2022 Jan 29;19(3):1536. doi: 10.3390/ijerph19031536 (PMC8835209; doi:10.3390/ijerph19031536)
Supplement: Supplementary file 1 [file ijerph-19-01536-s001.zip › ijerph-1493863-supplementary.pdf]

Focus group questions:

1) What are you currently using to manage your period pain and any other menstrual symptoms ? (probe for both pharmaceutical and non-pharmaceutical modalities such as heat and exercise).
2) How well are these working for you? (probe for effectiveness, and any barriers to usage such as cost)
3) What are the two or three most bothersome symptoms you get either just prior to or during your period ?
4) Have you ever previously used cannabis (medical or illicit) to manage any health-related conditions? (probe for stress, pain, mood disorders) If so can you tell us about your experiences (probe for negative effects as well as any positive, e.g anxiety or tachycardia)
5) If medicinal cannabis was available legally (from a medical practitioner) for period pain, would this be something you might consider using as part of your management strategy for period pain?
6) Does the use of medicinal cannabis raise any religious or cultural concerns that may impact the way your family or people in your community view or treat you?
7) How concerned are that that using a legal medicinal cannabis would limit your ability to drive, operate heavy machinery or impact your work if drug testing is conducted at your place of employment? Would this potentially limit your desire to want to use medicinal cannabis as a medical option for managing the symptoms of your period pain?
8) Are there any other barriers to using medicinal cannabis that you could think of (probe for other issues such as cost)
9) There are a number of ways that medicinal cannabis can be used, from inhaled (such as from a vaporiser) to taken orally (such as in a capsule) to used as pessary (inserted into the vagina). We would like to hear your thoughts on your preference for any of these options?
